# Attenuation of muscle spindle firing with artificially increased series compliance during stretch of relaxed muscle

**DOI:** 10.1113/EP090872

**Published:** 2023-10-19

**Authors:** Emily M. Abbott, Jacob D. Stephens, Surabhi N. Simha, Leo Wood, Paul Nardelli, Timothy C. Cope, Gregory S. Sawicki, Lena H. Ting

**Affiliations:** ^1^ Department of Biomedical Engineering Duke University Durham North Carolina USA; ^2^ Coulter Department of Biomedical Engineering Emory University and Georgia Institute of Technology Atlanta Georgia USA; ^3^ School of Physics Georgia Institute of Technology Atlanta Georgia USA; ^4^ School of Biological Sciences Georgia Institute of Technology Atlanta Georgia USA; ^5^ Woodruff School of Mechanical Engineering Georgia Institute of Technology Atlanta Georgia USA; ^6^ Department of Rehabilitation Medicine Emory University Atlanta Georgia USA

**Keywords:** muscle fascicle, proprioception, sensory feedback, tendon stiffness

## Abstract

Muscle spindles relay vital mechanosensory information for movement and posture, but muscle spindle feedback is coupled to skeletal motion by a compliant tendon. Little is known about the effects of tendon compliance on muscle spindle feedback during movement, and the complex firing of muscle spindles makes these effects difficult to predict. Our goal was to investigate changes in muscle spindle firing using added series elastic elements (SEEs) to mimic a more compliant tendon, and to characterize the accompanying changes in firing with respect to muscle–tendon unit (MTU) and muscle fascicle displacements (recorded via sonomicrometry). Sinusoidal, ramp‐and‐hold and triangular stretches were analysed to examine potential changes in muscle spindle instantaneous firing rates (IFRs) in locomotor‐ and perturbation‐like stretches as well as serial history dependence. Added SEEs effectively reduced overall MTU stiffness and generally reduced muscle spindle firing rates, but the effect differed across stretch types. During sinusoidal stretches, peak and mean firing rates were not reduced and IFR was best‐correlated with fascicle velocity. During ramp stretches, SEEs reduced the initial burst, dynamic and static responses of the spindle. Notably, IFR was negatively related to fascicle displacement during the hold phase. During triangular stretches, SEEs reduced the mean IFR during the first and second stretches, affecting the serial history dependence of mean IFR. Overall, these results demonstrate that tendon compliance may attenuate muscle spindle feedback during movement, but these changes cannot be fully explained by reduced muscle fascicle length or velocity, or MTU force.

## Abstract (highlights)

- **What is the central question of the study?**
  Little is known about the effects of tendon compliance on muscle spindle function: does increasing the series compliance of the muscle–tendon unit reduce Ia afferent responses to stretch? And what is the relationship between muscle spindle firing rates and muscle fascicle biomechanics?
- **What is the main finding and its importance?**
  Muscle spindle firing was generally attenuated with added series compliance, with the exception of firing rates during sinusoidal stretches. Overall, the changes depended upon stretch profiles, and could not be fully explained by muscle fascicle length or velocity, or muscle–tendon unit force.

## 1 INTRODUCTION

Muscle spindle sensory organs provide critical mechanosensory information for movement, but as muscle spindles are intramuscular sensors, the coupling of muscle spindle feedback to joint motion may depend on the mechanical properties of the muscle–tendon unit (MTU) (Blum et al., 2019; Maas & Lichtwark, 2009), which can change in ageing and disease (Khor et al., 2021; Lindemann et al., 2020; Onambele et al., 2006; Stenroth et al., 2012). Muscle spindle sensory feedback contributes to the control of movement and maintenance of posture (Abelew et al., 2000; Klint et al., 2008; Lockhart & Ting, 2007; Matthews, 1964; Mayer & Akay, 2021; Rothwell et al., 1982; Sanes et al., 1985). Muscle spindle primary afferents fire reliably to stretch of the MTU, particularly when the muscle is in a relaxed state, and fire in relation to both the speed and the extent of the movement. For example, during locomotion, the MTU is stretched and shortened periodically at a relatively low frequency and muscle spindles exhibit approximately sinusoidal firing rates (Boyd, 1976; Prochazka & Gorassini, 1998a, 1998b; Prochazka et al., 1977). In contrast, during discrete perturbations muscles can be very rapidly stretched, and muscle spindles exhibit very high and transient firing rates with various components that are sensitive to the acceleration, velocity and/or displacement of the entire MTU (Gottlieb & Agarwal, 1979; Honeycutt et al., 2012; Matthews, 1963; Schäfer, 1967). As MTU stretch reflects the sum of the displacement of the muscle compartment and of the free tendon that is mechanically in‐series with the muscle, increased compliance (length change with respect to force) of the tendon will reduce the displacement of the muscle for a given displacement of the MTU. Thus, muscle spindle sensory organs throughout the belly of the muscle are only indirectly coupled to skeletal motion through the compliant tendon.

Discerning the effects of increased tendon compliance on muscle spindle firing during behaviour in either humans or animals is extremely difficult. There is mounting evidence that tendons become more compliant in older adults (Svennson et al., 2016; Lindemann et al., 2020; Mademli & Arampatzis, 2008; Onambele et al., 2006; Stenroth et al., 2012). Accordingly, older adults have decreased proprioceptive ability that affects their movement and posture (Henry & Baudry, 2019; Horak et al., 1989; Petrella et al., 1997; Skinner et al., 1984). However, ageing studies cannot isolate the effects of tendon compliance from other physiological effects of age, such as changes to the muscular and nervous systems, and directly manipulating tendon compliance in vivo is highly invasive and could alter sensorimotor control strategies. Most recordings from animal muscle spindle afferents occur in acute, anaesthetized preparations such as the rat, where MTU stretch is directly controlled and muscle spindle afferent firing is recorded from the dorsal roots of the spinal cord. Prior work has examined the potential consequences of tendon compliance on spindle behaviour during imposed stretches that mimicked the locomotor cycle in the cat (Elek et al., 1990). However, this work indirectly inferred series compliance based on spindle firing, rather than altering compliance and observing direct consequences on spindle firing, and without direct fascicle length measurements. Acute studies in aged rats do show that the muscle tendon unit must be stretched farther to initiate firing of action potentials (Miwa et al., 1995), but age may also affect mechanical properties of aponeuroses and of the muscle itself, and age‐related changes in animal models are different from in humans (Kjaer, 2004), limiting direct comparisons. Thus, it is critical to directly manipulate series compliance without altering the myotendinous structure.

The relationship between spindle afferent firing responses and muscle fascicle biomechanics is still an open question. Prior interpretations of muscle spindle firing behaviour have largely relied on its association with measurements of the MTU, rather than measurements of the muscle fascicle. However, forces and displacements at the level of the spindle are indirectly coupled to the motion of the skeleton via the series elasticity of the tendon (Hoffer et al., 1989; Maas & Lichtwark, 2009). Further, the forces carried by the tendon are shared between the muscle fascicles and the extracellular matrix such that the fascicle displacement cannot be easily inferred from MTU mechanics. Evidence from measures of human muscle spindle firing shows a close relationship between muscle spindle firing and muscle fascicle stretch in slow, postural‐sway‐like stretches (Day et al., 2017). However, there are few studies concurrently recording fascicle length and muscle spindle firing.

It is difficult to determine how series compliance will affect muscle spindle behaviour due to mechanical interactions at the level of the spindle and the unique firing responses of spindles to various stimuli. In rapid stretches, muscle spindles exhibit initial bursts at the onset of stretch (Blum et al., 2017, 2019, 2020; Hunt & Ottoson, 1976; Proske & Stuart, 1985; Schäfer, 1967; Schäfer & Kijewski, 1974), which are followed by a dynamic response in which firing rates increase with muscle stretch (Haftel et al., 2004). When held at a new longer length, muscle spindle firing rates decrease rapidly and then slowly decrease to a new steady‐state firing rate (Boyd, 1976). Even though muscle spindle firing has been shown to closely follow MTU velocity in active locomotion in cats (Prochazka & Gorassini, 1998a, 1998b) and fascicle length and velocity in small sinusoidal stretches in humans (Day et al., 2017), it has also been shown to scale non‐linearly with MTU stretch amplitude in cats (Hunt & Ottoson, 1977; Matthews & Stein, 1969). While series compliance would likely attenuate all these firing behaviours by dampening muscle stretch, it is especially difficult to predict how different response properties of muscle spindles will be affected.

Both the discharge patterns of muscle spindles and the mechanical characteristics of extrafusal and intrafusal muscle fibres, are contingent upon preceding movement and activation events, properties generically referred to as ‘history dependence’. Repeated stretches at a short intervals reveal an absence of the initial burst and lower dynamic response of muscle spindles in succeeding stretches (Blum et al., 2017, 2019, 2020; Hunt & Ottoson, 1976; Nichols & Cope, 2004; Proske & Stuart, 1985). In the context of this manuscript, the term ‘history dependence’ thus denotes variations in muscle spindle discharge during successive cycles of muscle stretches that have been attributed to history‐dependence of transient intrafusal muscle fibre stiffness in response to stretch perturbations (Nichols & Cope, 2004). It should be noted, however, that in the context of muscle physiology, ‘history dependence’ can also be used to denote the effect of muscle length changes on steady state force and stiffness, i.e. residual force enhancement or depression (Abbott & Aubert, 1951; Edman et al., 1982; Joumaa et al., 2012; Nishikawa, 2016). It is important to note that these definitional distinctions here neither exclude nor encompass hypotheses that explore the causal relationship between the history‐dependent properties of muscle and their spindles.

Our goal was to directly manipulate series compliance and to observe the immediate effects on muscle spindle firing during stretch. We hypothesized that increased tendon compliance (or decreased stiffness) would reduce muscle spindle firing during stretch. We further hypothesized that muscle spindle firing would be more closely related to muscle fascicle versus MTU stretch.  We experimentally increased series compliance and characterized the effects on muscle spindle afferent firing during MTU stretch of relaxed muscle while recording muscle spindle firing and muscle fascicle length. Effective series compliance in anaesthetized animals was increased by adding series elastic elements (SEEs) to the end of the MTU. The same sinusoidal, ramp–hold–release, and repeated triangular stretches were applied to the MTU and to the MTU+SEE complex. Simultaneously, we recorded muscle spindle firing from identified Ia afferents, muscle fascicle length using sonomicrometry and MTU force. Given the non‐linear relationship between MTU stretch amplitude and muscle spindle firing in sinusoidal stretches, we tested whether potential changes in muscle spindle firing could be accounted for by changes in fascicle displacement and velocity between control and SEE groups. In ramp–hold–release stretches, we compared the relationships of muscle spindle firing during the hold phase to fascicle displacement and force, as the decrease of muscle spindle instantaneous firing rate (IFR) during the hold phase has been speculated to be driven by fascicle length changes. In triangular stretches, we tested whether the stretch response after the first stretch could be attributed to changes in fascicle displacement or MTU force.

## 2 METHODS

### 2.1 Ethical approval

All procedures and experiments were approved by the Georgia Institute of Technology Institutional Animal Care and Use Committee (protocol number A18042).

### 2.2 Animal care

Adult female Wistar rats (250–300 g; Charles River Laboratories, Wilmington, MA, USA) were studied in terminal experiments only and were not subject to any other experimental procedures. All animals were housed in clean cages and provided food and water ad libitum in a temperature‐ and light‐controlled environment in the Georgia Institute of Technology Physiological Research Laboratory. Following data collection, animals were killed by exsanguination.

Surgery was carried out in the same manner described in previous experiments from this laboratory (Vincent et al., 2017). In brief, rats were deeply anaesthetized (complete absence of withdrawal reflex) by inhalation of isoflurane, initially in an induction chamber (5% in 100% O_2_) and for the remainder of the experiment via a tracheal cannula (1.5−2.5% in 100% O_2_). Vital signs were continuously monitored including core temperature (36–38°C), $P_{{\mathrm{CO}}_{2}}$ (3%–5%), respiratory rate (40−60 breaths/min), pulse rate (300‐450 bpm) and $S_{{\mathrm{pO}}_{2}}$ (>90%). Anaesthesia level and vital signs were maintained by adjusting isoflurane concentration, radiant and water‐pad heat sources, and by scheduled subcutaneous injection of saline (1 ml/h subcutaneous). Surgical preparation followed by data collection lasted up to 8 h.

### 2.3 Data collection

**FIGURE 1:**
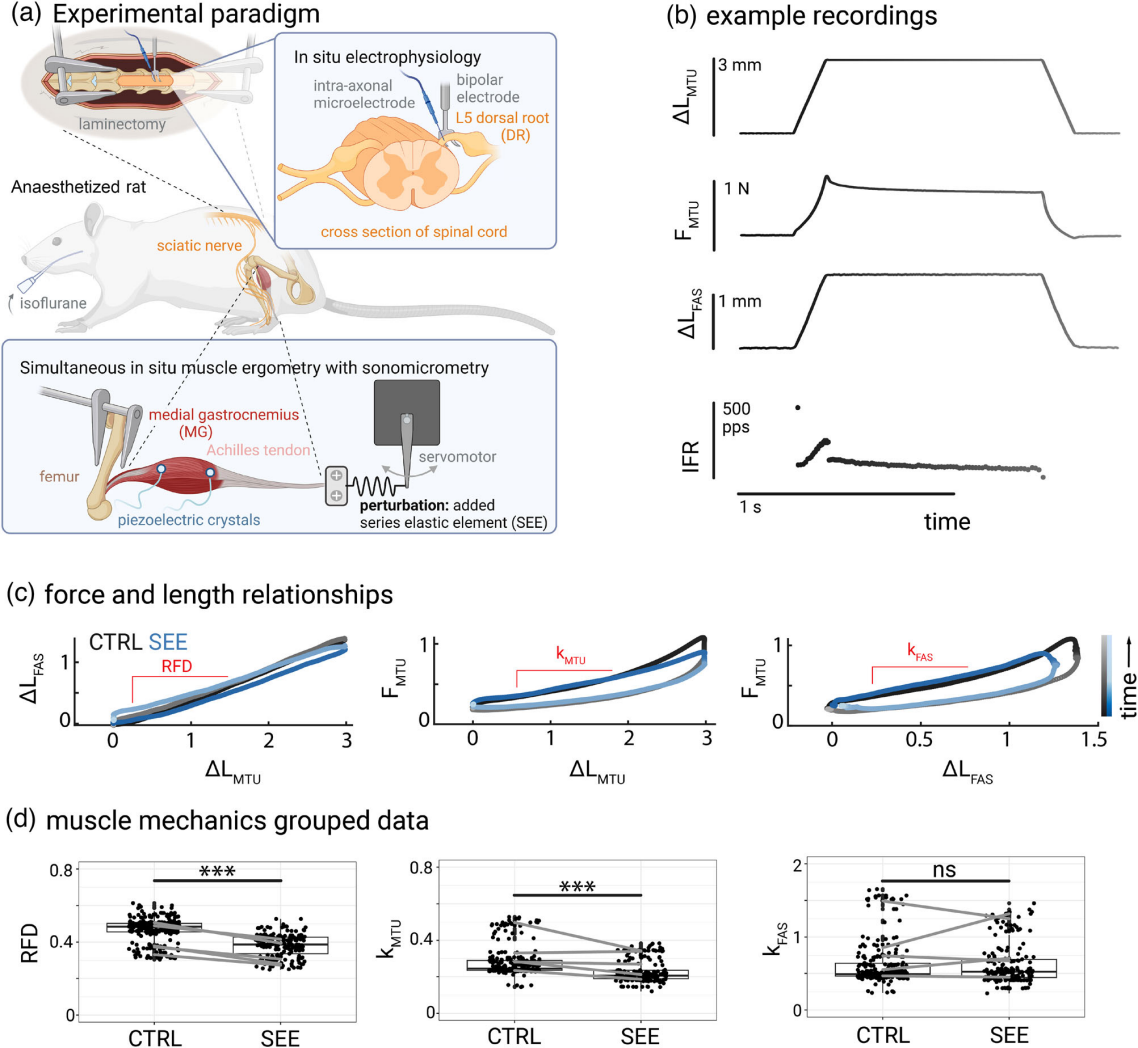
Experimental design, sample data and stiffness analysis with added SEE. (a) Under isoflurane anaesthesia, the L5 dorsal root was suspended on a bipolar hook electrode while a microelectrode was used to intracellularly record the activity of single cells. The femur was also clamped in place, the medial gastrocnemius (MG) was dissected away from the rest of the triceps surae, and the Achilles tendon was fixed either directly to the servomotor or with a series elastic element (SEE) between the tendon and motor. Piezoelectric crystals were also inserted into the MG along a single fascicle and sutured into place (created with BioRender.com). (b) Example recordings of MTU length change, MTU force, fascicle length change and muscle spindle IFR during ramp–hold–release stretches. (c) Examples of fascicle displacement vs MTU displacement, MTU force versus MTU displacement, and MTU force vs fascicle displacement in example control (CTRL) and SEE trials. Line colours correspond to time in order to display differences in lengthening and shortening, corresponding to the shading in (b). Labelled red lines represent the metrics analysed in (d). (d) Grouped metrics show reduced relative fascicle displacement (RFD) by 0.083 ± 0.004 mm/mm (mean ± SEM, *P* < 1 × 10^−15^, *n* = 441 from five animals), reduced MTU stiffness (*k*_MTU_) by 0.050 ± 0.004 N/mm (mean ± SEM, *P* < 2 × 10^−16^), and no change in fascicle stiffness (*k*_FAS_) at 0.5 N in ramp stretches (*P* = 0.390). Box plots indicate the mean and first and third quartiles, grey lines indicating change determined by linear regression for each animal. ***P < 0.001; ns, no significance.

To examine the effects of tendon compliance on muscle spindle behaviour, we added elastic elements to the end of the medial gastrocnemius MTU to increase the series compliance of the MTU, mimicking a more compliant tendon. In SEE conditions, ‘MTU’ refers to the MTU and SEE complex as one unit. Data collection was conducted in a similar manner as has been described in previous work from this laboratory (Vincent et al., 2017). Sinusoidal stretches were applied at 2 mm amplitude at 2 Hz, ramp–hold–release stretches at 3 mm length change at a velocity of 20 mm/s, and triangular stretches consisted of three repetitions of 3 mm length changes at 3.5 mm/s. MTU length and force were measured from a servomotor (Aurora Scientific (Aurora, Ontario, Canada) 310C‐LR), and muscle fascicle length was measured with sonomicrometry crystals (Sonometrics (London, Ontario, Canada)) implanted along the same muscle fascicle within the MG, all sampled at 17.8 kHz (Figure 1a,b). Recordings from spindle afferents were taken intracellularly via a glass microelectrode inserted into dorsal root afferents. Ventral root motor efferents were also stimulated during these experiments, but the current analysis only considers passive stretches. Sinusoidal stretches also incorporated ventral root stimulation after several passive cycles, and only the initial passive cycles are analysed here. Data were collected and stored using Cambridge Electronic Design (CED; Cambridge, UK) Power 1401 and Spike2 software and exported to MATLAB (The MathWorks Inc., Natick, MA, USA) for analysis. Statistical comparisons were performed using R software (R Foundation for Statistical Computing, Vienna, Austria).

### 2.4 Data processing

Sonomicrometry readings were calibrated by recording channel voltage and measuring the distance between the probes in the muscle at resting length and at 1 mm of MTU displacement with no SEE. Measurements of motor position, motor force and fascicle displacement were first down‐sampled by a factor of 20 to approximately 900 Hz, then lowpass filtered with a fourth‐order Butterworth filter with a 100 Hz cutoff. Then data were smoothed and differentiated with a second‐order Savitsky–Golay filter with a window width of 21 samples (approximately 24 ms). Sonomicrometry measurements were calibrated by measuring the length between the two crystals along the muscle fascicle corresponding to two measurements, in order to match voltage values from the data channel to physical lengths. The calibration values were collected from all animals, and the mean calibration factor (in mm/V) was used as the scaling factor for all trials as little variability has been observed between experiments.

### 2.5 Data analysis

To validate the effects of SEEs, the length change of the fascicle relative to the MTU, the stiffness of the MTU, and the stiffness of the fascicle were computed for each stretch type. Stiffness was computed by fitting a second‐order polynomial to force/length curves at 0.5 N in ramp–hold–release stretches, and 0.4 N in triangles and sinusoids, as the lower velocities in triangles and lower amplitudes and velocities in sinusoids elicited less force than in ramp–hold–release stretches. Stiffness (in N/mm) was calculated by computing the slope of the tangent line at these force thresholds.  The relative fascicle displacement (RFD) was estimated in the same manner, estimating the slope of the tangent line (d*L*_Fas_/d*L*_MTU_) at 0.5 or 0.4 N (Figure 1c,d).

**FIGURE 2:**
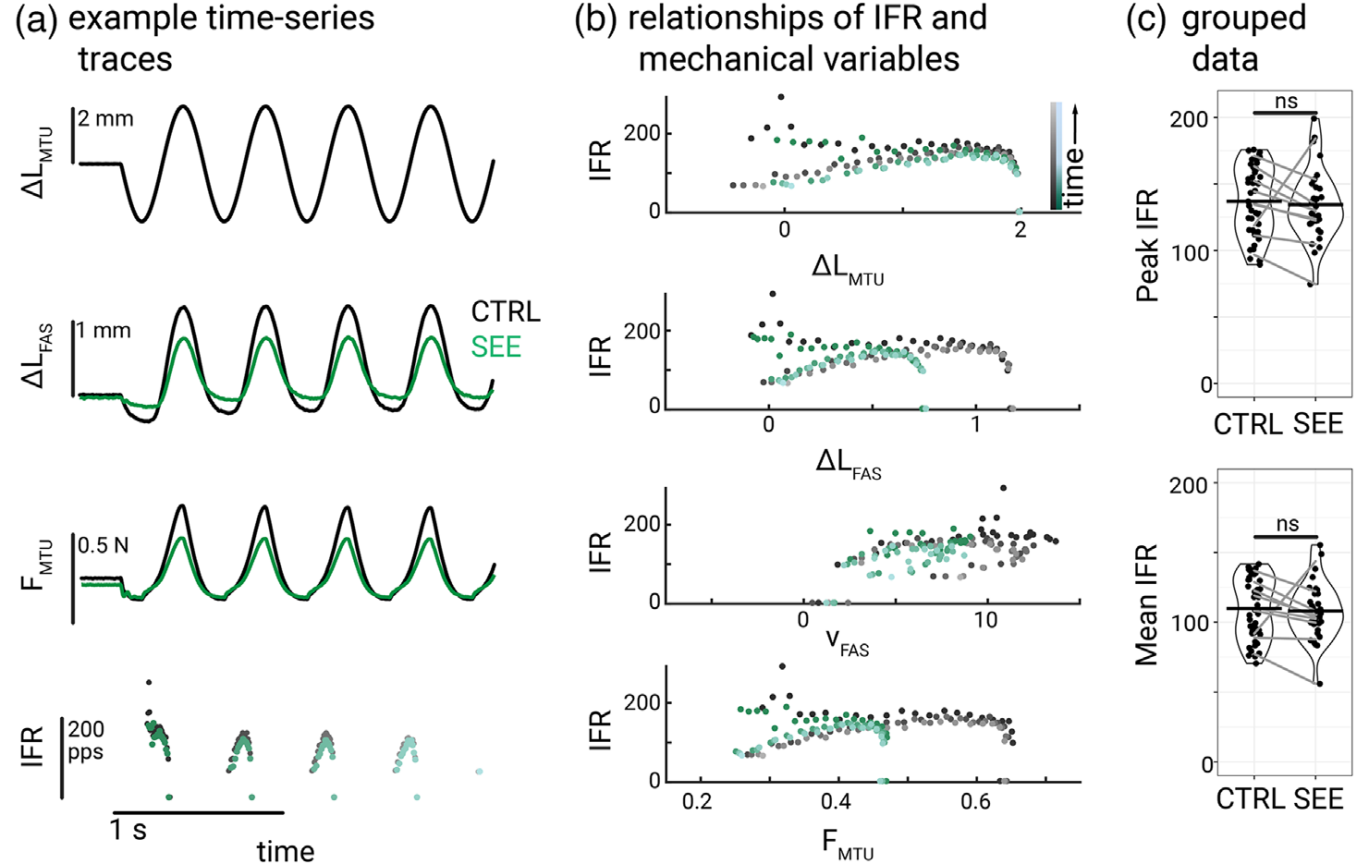
Changes in firing rates and relationships to muscle and MTU mechanics during 2 mm, 2 Hz sinusoidal stretches of the MTU. (a) Addition of SEE revealed reduced fascicle displacement in both shortening and lengthening, reduced MTU force and reduced muscle spindle IFR (control, black; SEE, green; darker points appearing earlier in time). (b) Relationships between IFR and MTU displacement, fascicle displacement and velocity, and MTU force reveal the strongest relationship between IFR and fascicle velocity (*R* ^2^ = 0.38 ± 0.16). Initial burst is evident in both control and SEE. (c) Statistical comparisons of peak and mean IFR after the first cycle reveal no significant reduction in peak IFR (peak IFR *P* = 0.176, *n* = 77 from 9 afferents) and no reduction in mean IFR (*P* = 0.226) with added SEE. Horizontal black bars indicate sample means and grey lines represent the change for each afferent determined by linear regression. ns, no significance.

**FIGURE 3:**
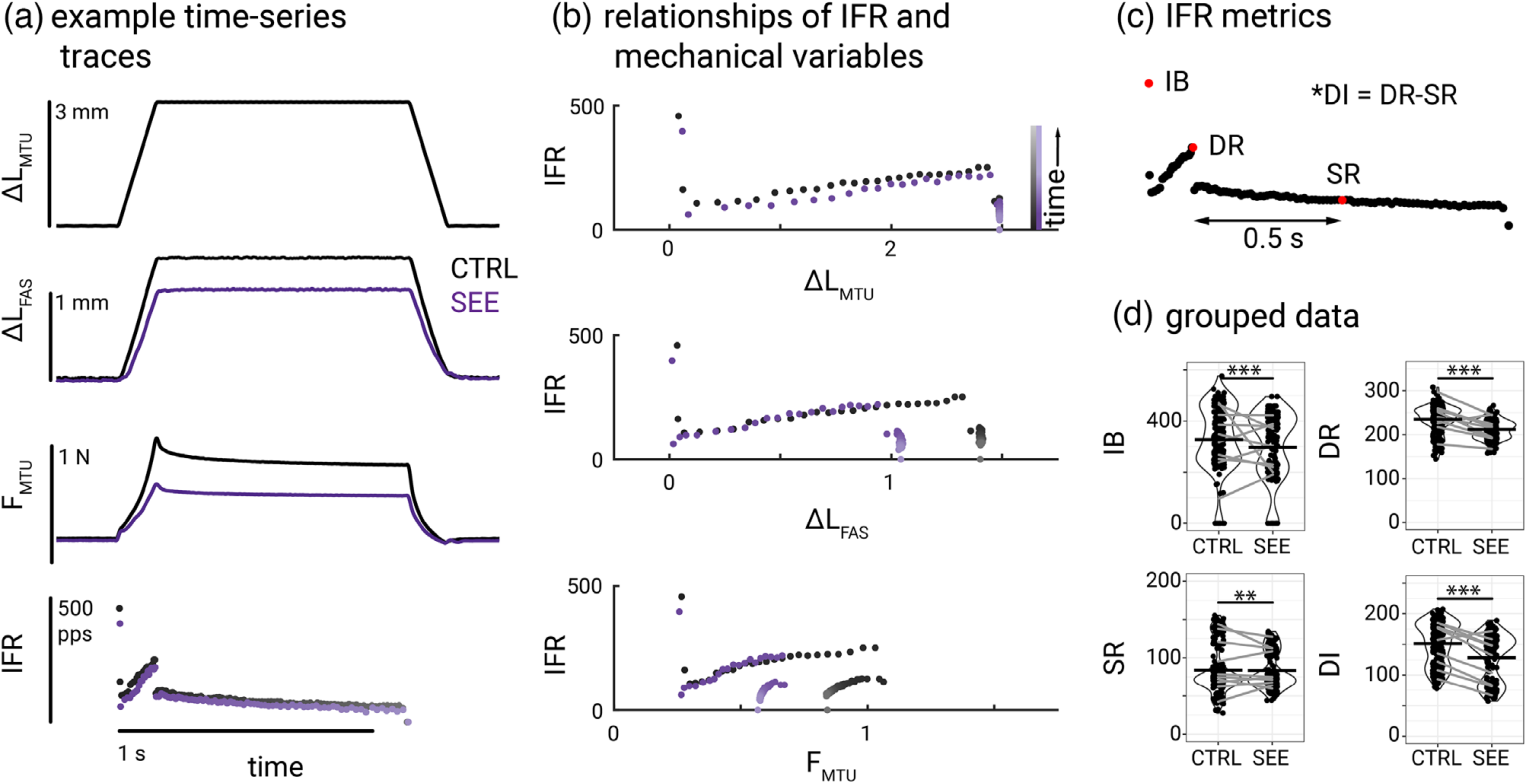
IFR versus muscle and MTU mechanics during ramp–hold–release stretches. Stretches of 3 mm, 20 mm/s were applied to the MTU, followed by a 1 s hold phase and subsequent release. (a) Muscle fascicle displacement and MTU force were subsequently reduced with added SEE (black, CTRL; purple, SEE). (b) Relationships between IFR and MTU displacement, fascicle displacement, and MTU force reveal none of these variables predict initial burst or dynamic response, but MTU force shows a concomitant decrease with the decrease in IFR during the hold phase. (c) Examples of initial burst (IB), dynamic response (DR), static response (SR) measured 0.5 s after the DR, and dynamic index (DI) as the difference in the dynamic and static responses. (d) Across trials, SEE resulted in a 47.5 ± 10.1 pps decrease in initial burst (*P* < 1 × 10^−5^, *n* = 441 from 10 afferents across 5 animals), 28.5 ± 1.5 pps decrease in DR (mean ± SEM, *P* < 2 × 10^−16^), 3.2 ± 1.0 pps decrease in SR (mean ± SEM, *P* = 1.59 × 10^−3^), and 25.3 ± 1.3 pps decrease in DI (mean ± SEM, *P* < 2 × 10^−16^). Horizontal black bars indicate sample means and grey lines represent the change for each afferent determined by linear regression. **P < 0.01, ***P < 0.001; ns, no significance.

During sinusoidal stretches, peak firing rate and mean firing rate were analysed following the first cycle to analyse changes in spindle responses at a steady state and ignore history‐dependent spindle firing in the first cycle (Figure 2c). During ramp‐and‐hold stretches, initial bursts, dynamic responses and static responses were extracted from firing rates (Figure 3c). If there was no initial burst discernible from the firing response during lengthening, the initial burst value for the particular stretch was set to 0. The dynamic response was taken as the peak firing rate at the end of lengthening. The static response was taken as the firing rate approximately 0.5 s into the hold phase, and the dynamic index is the difference between the dynamic and static responses. During triangular stretches, mean firing rate and spike counts were computed for the first and second stretches, along with initial burst. History dependence was analysed by comparing the differences in mean IFR and spike count between the first and second stretches for each trial. Linear regression analyses were performed only after the first stretches in sinusoids and triangles, and only on the static response in ramp–hold–release stretches, as forces and length measurements are not expected to predict the more dynamic components of firing responses.

Trials were excluded according to IFR and force recordings. Individual stretches where action potentials could not be discriminated from the cell recording were excluded. Additionally, recordings from one cell were excluded as force records had a signal to noise ratio of less than 2.

Statistical comparisons of firing and stiffness were conducted with linear mixed models in R, using the *lmer* functionality of the *lmerTest* package (Kuznetsova et al., 2017). In total, data from eight animals were analysed based on availability in control and SEE conditions: 441 ramp‐and‐hold stretches from 10 afferents across five animals, 120 triangular stretches from 11 afferents across five animals, and 77 sinusoidal stretches from nine afferents across four animals. In stiffness and fascicle displacement analyses, the effects of tendon compliance were determined by linear mixed models, with individual animals serving as random effects and SEE as a fixed effect. In firing rate and regression model comparisons, individual afferents served as random effects and SEE as a fixed effect. Linear regressions were computed in MATLAB, and per‐trial *R* ^2^ values were exported and analysed in R. Reported values are the mean ± SD unless stated otherwise.

## 3 RESULTS

Overall, adding the SEE reduced fascicle displacement relative to MTU displacement during stretch and reduced effective tendon stiffness (Figure 1c). Relative fascicle displacement was reduced in the SEE condition by 0.083 ± 0.004 mm/mm (mean ± SEM) in ramps (*P* < 1 × 10^−15^, 441 trials across 5 animals), 0.083 ± 0.009 mm/mm in triangles (*P* < 1 × 10^−13^, 120 trials across 5 animals), and 0.057 ± 0.01 mm/mm (mean ± SEM) in sinusoids (*P* < 1 × 10^−5^, 77 trials across 4 animals). MTU stiffness was reduced by 0.050 ± 0.004 N/mm (mean ± SEM, *P* < 2 × 10^−16^) in ramps, 0.022 ± 0.005 N/mm in triangles (*P* < 1 × 10^−4^) and 0.028 ± 0.0050 N/mm (mean ± SEM, *P* < 2 × 10^−7^) in sinusoids. In contrast, there were no differences in fascicle stiffness with added SEE across conditions (all *P* > 0.122). Note that limiting study to female rats precluded examination of potential sex difference.

During sinusoidal stretches, adding the SEE did not reduce muscle spindle firing rates (see exemplar in Figure 2a). There was no reduction in mean firing rate with added SEE (*P* = 0.226, *n* = 77 from 9 afferents across 4 animals), or in peak firing rate (*P* = 0.176) (Figure 2c). In some cases, greater firing responses were observed in the first cycle (Figure 2). IFR was generally poorly correlated with muscle fascicle length after the first stretch (*R* ^2^ = 0.05 ± 0.04), and the regression slope was reduced in SEE conditions by 39.0% (8.93 ± 2.76 pps/mm, mean ± SEM, *P* = 1.87 × 10^−3^). IFR was best correlated with fascicle velocity (*R* ^2^ = 0.38 ± 0.16), but the regression slope was increased by 32.7% in SEE conditions (control rate = 6.84 ± 0.56, change = 2.24 ± 0.37 pps/mm/s, mean ± SEM, *P* < 1 × 10^−7^). Additionally, MTU force was weakly correlated with muscle spindle firing rate (*R* ^2^ = 0.10 ± 0.09) but did not show a difference in regression slope (*P* = 0.270) or intercept (*P* = 0.227) between control and SEE conditions.

During ramp‐and‐hold stretches, adding the series elastic element decreased the initial burst at the onset of stretch, during the ramp, and during the hold phase. Fascicle length changes were similar to MTU changes, but with reduced amplitude with added SEE (see Figure 3a for example). The MTU force rose faster than the ramp displacement and gradually decreased during the hold period. At the onset of the ramp, muscle spindle firing exhibited an initial burst that was reduced by the added SEE (control rate = 333 ± 29 pps, change = 47.5 ± 10.1 pps, mean ± SEM, *P* < 1 × 10^−5^, *n* = 441 from 10 afferents across 5 animals). The dynamic response of the muscle spindle during the ramp phase (3 mm, 20 mm/s), was attenuated with SEE by 28.5 ± 1.5 pps (mean ± SEM, *P* < 2 × 10^−16^). The static firing rate during the hold phase also decreased with added SEE by 3.2 ± 1.0 pps (mean ± SEM, *P* = 1.59 × 10^−3^). Accordingly, the dynamic index, indicating the decrease in firing rate during the hold phase, was also reduced with added SEE by 25.3 ± 1.3 pps (mean ± SEM, *P* < 2 × 10^−16^).

During the hold phase, muscle spindle IFR was strongly correlated with MTU force (*R* ^2^ = 0.72 ± 0.14), and weakly correlated with fascicle displacement and velocity and MTU displacement and velocity (all mean *R* ^2^ between 0.06 and 0.34). During the hold phase, muscle fascicle displacement did not predict the observed changes in muscle spindle firing rate. As muscle spindle firing decreased, the muscle fascicle lengthened (Figure 3b) and linear regression analysis revealed a negative relationship between IFR and fascicle displacement that changed with SEE (slope = −1294 ± 157, change = 135.6 ± 49.7, mean ± SEM, *P* = 6.66 × 10^−3^). Positive relationships were found between IFR and MTU force in control (slope = 369 ± 167, *R* ^2^ = 0.75 ± 0.12) and SEE trials (slope = 643 ± 361, *R* ^2^ = 0.69 ± 0.16), but the slopes of these fits were different between control and SEE groups (*P* < 2 × 10^−16^).

**FIGURE 4:**
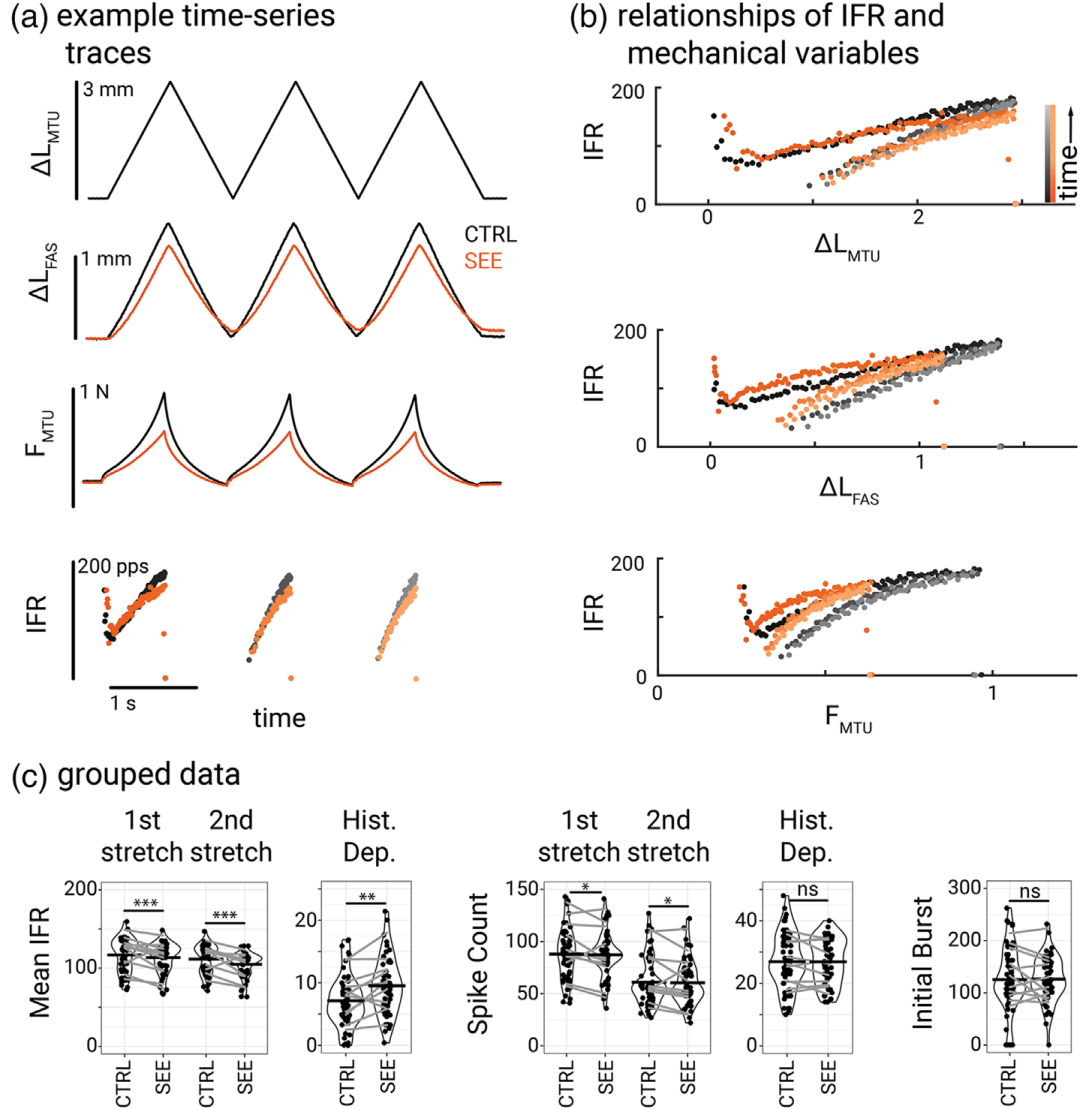
Relationships of MTU displacement and force and fascicle displacement to muscle spindle IFR during triangular stretches. Effects of series elasticity on muscle fascicle displacement, MTU force and muscle spindle IFR repeated triangle stretch trials of 3 mm, 3.5 mm/s. (a) Muscle fascicle displacement and MTU force were reduced with added SEE (black, CTRL; orange, SEE). In both conditions, stretches evoked IBs and DRs in the first stretch (black, CTRL; dark orange, SEE) that were absent in subsequent stretches (grey, CTRL; light orange, SEE). (b) Relationships between IFR and MTU displacement, fascicle displacement and MTU force. All variables reasonably predict IFR during the second and third stretches, but not the IB or DR of the first stretch. (c) Across groups, SEE resulted in no change in IB (*P* = 0.458). Spike counts were reduced during the first stretch by 5.6 ± 2.4 spikes (mean ± SEM, *P* = 0.0198, *n* = 120 from 11 afferents) and in the second stretch by 4.1 ± 1.8 (mean ± SEM, *P* = 0.0266). Mean IFR was reduced in the first stretch by 8.1 ± 2.4 pps (mean ± SEM, *P* = 9.54 × 10^−4^) and in the second stretch by 10.4 ± 2.2 pps (mean ± SEM *P* < 1 × 10^−5^). Subsequently, mean IFR history dependence was increased by 2.4 ± 0.8 pps (mean ± SEM, *P* = 3.16 × 10^−3^), but spike count history dependence was not (*P* = 0.157). Horizontal black bars indicate sample means and grey lines represent the change for each afferent determined by linear regression. *P < 0.05, **P < 0.01, ***P < 0.001; ns, no significance.

During repeated triangular stretches, SEE decreased firing rates and increased the history dependence of muscle spindle mean IFR. At the onset of the first stretch, muscle spindles produced initial bursts in both conditions as well as a response during the ramp stretch. In the second and subsequent stretches, the initial burst was absent, and the response during lengthening was reduced (Figure 4a,b). During the first stretch, SEE had no effect on initial bursts (*P* = 0.458, *n* = 120 from 11 afferents across 5 animals) but did reduce the mean IFR by 8.2 ± 2.4 pps (mean ± SEM, *P* = 9.54 × 10^−4^, Figure 4c) and the spike count by 5.6 ± 2.4 spikes (mean ± SEM, *P* = 0.0198). In the second stretch, SEE similarly reduced spike counts by 4.1 ± 1.8 spikes (mean ± SEM, *P* = 0.0266) and reduced the mean IFR by 10.4 ± 2.2 pps (mean ± SEM, *P* < 1 × 10^−5^). In the same manner, the history dependence of spike counts was not affected by SEE, but the history dependence of mean IFR was increased with added SEE by 2.4 ± 0.8 pps (mean ± SEM, *P* = 3.16 × 10^−3^).

During the second and third stretches, muscle spindle IFR was moderately correlated with MTU force (*R* ^2^ = 0.53 ± 0.08), MTU displacement (*R* ^2^ = 0.51 ± 0.11), and fascicle displacement (*R* ^2^ = 0.48 ± 0.13), and not correlated with MTU nor fascicle velocity (*R* ^2^ = 0.15 ± 0.06 and 0.12 ± 0.11, respectively). Interestingly, the relationship between IFR and fascicle displacement during the second and third stretches did not change with SEE (*P* = 0.138), but the relationships to MTU length and force did change (*P* < 1 × 10^−15^ and *P* < 1 × 10^−6^, respectively). However, overall, the relationships between muscle spindle IFR with respect to all of the biomechanical variables were not unique and changed between the first and second stretches (Figure 4b).

## 4 DISCUSSION

Our data show that increasing the effective series compliance decreased the firing rate in muscle spindles during muscle stretch. Reductions in firing rates with added series elasticity depended on the muscle stretch profile, with reduced firing rates in ramp‐and‐hold stretches and in repeated triangular stretches. These data suggest that increased tendon compliance could reduce mechanosensory feedback during movement. Interestingly, the peak and mean firing rates during sinusoidal stretches did not significantly decrease with increased compliance, and the initial burst in triangular stretches was not significantly reduced as in ramp‐and‐hold stretches. Notably, although muscle spindle firing was related to fascicle displacement and velocity during continuous stretches (sinusoids, second and third triangles), it had non‐unique relationships to fascicle displacement during ramp stretches, and negative relationships to fascicle length during the hold phase after a ramp. Our data show that fascicle length does not completely explain the nuances of muscle spindle behaviour, and that more complex models may be necessary to understand how changes in mechanical properties of connective tissues affect sensory feedback from muscle spindles.

The experiments presented here validated the use of adding series springs to experimentally increase tendon compliance and decouple biomechanical relationships within the MTU. Using sonomicrometry revealed that the relative stretch in muscle fascicles with respect to the effective MTU was reduced, indicating increased stretch in the effective tendon. Further, total muscle fascicle stiffness remained unchanged while total MTU stiffness was reduced when adding SEEs. Thus, this experimental design effectively increased the compliance of the compartment mechanically in series with the muscle without disturbing the properties of the muscle itself, isolating the effects of series elasticity. At longer stretch lengths, the difference in the MTU force and length was larger, as the non‐linear elasticity of the muscle tissues was engaged. Thus, increasing series compliance changes the distribution of length (and force via stiffness) within the compartment of the MTU enabling experiments to probe the role of different biomechanical signals (e.g. length, velocity; force, yank) in shaping muscle spindle output. A limitation of the current experiment is that, in the added SEE condition, *both* MTU force and fascicle length decreased, making it difficult to clearly distinguish the roles of force versus length‐related dynamics as driving inputs of muscle spindle firing. The lack of change in firing rates in sinusoidal stretches despite decreases in fascicle displacement and MTU force with added SEEs demonstrates such.

In sinusoidal stretches, muscle spindle firing was not well‐correlated with any of the tested biomechanical variables, and some relationships differed between control and SEE trials. Contrary to prior work (Day et al., 2017), muscle fascicle displacement and velocity were not well correlated with muscle spindle firing during sinusoidal stretches. However, this prior work incorporated phase shifts between the measured IFR and displacement and velocity which were not included here. While correlations were not strong, the change in regression coefficients is still indicative of a more complex relationship to muscle fascicle biomechanics than simply displacement or velocity. Further, while MTU force showed a poor correlation, there was no evidence of an effect of added compliance (SEE) on this relationship. However, the relationships of IFR to any of these variables is markedly complex (Figure 2b), and thus a more rigorous model comparison is warranted to further test the relationships of muscle spindle firing to fascicle mechanics. Another limitation is the assumed series arrangement of the muscle and tendon, as muscles and tendons are not always completely in series mechanically (for review see Herzog, 2019). Muscles and tendons can be considered mechanically in series when there is a considerable length of free tendon, as is the case with the MG and Achilles tendon analysed here. Thus, the addition of series elasticity mimics an increase in tendon compliance in our experimental preparation (Figure 1). It is important to note that our findings regarding series compliance per se may not apply to muscles with more complex myotendinous geometries. Yet while complex myotendinous geometries may pose a challenge for estimating differences in fascicle stretch with increased series compliance, this experimental design addressed this by directly measuring from fascicles.

The change in muscle spindle firing during ramp‐and‐hold stretches suggests that increased series compliance may affect reflexive control responses in the muscle. The monosynaptic stretch reflex is driven by primary muscle spindle afferent firing during rapid muscle stretch, and our data show that the responses of these primary afferents are reduced with increased series compliance. Further, supraspinal reflex pathways, such as those evoked during balance perturbations, may also be diminished by this reduction in sensory feedback from primary muscle spindle afferents (Lockhart & Ting, 2007; Welch & Ting, 2008; Welch & Ting, 2009). Although initial bursts were not significantly reduced in triangular stretches, the data set of triangular stretches had a smaller sample size and initial bursts were elicited less reliably than in ramp‐and‐hold stretches.

The negative relationships between muscle spindle firing and fascicle length at constant MTU length may be due to the stress relaxation of extrafusal skeletal muscle fibres and intrafusal muscle spindle fibres after being stretched. It has been suggested that the slowing of muscle spindle firing during the hold phase is due to fascicle shortening, but fascicle displacement *increased* during the hold phase of ramp–hold–release stretches as firing decreased (Figure 3b). Prior model predictions also showed that changes in muscle fascicle length are in opposition to muscle spindle firing rates during conditions where MTU length does not predict muscle spindle firing (Blum et al., 2017). While the relationship between IFR and MTU force was different in SEE trials, it did predict the concomitant decrease in IFR. It has been suggested that the decrease of muscle spindle firing during the hold phase is due to the stress relaxation of intrafusal muscle spindle fibres (Boyd, 1976; Nichols & Cope, 2004). As both extrafusal and intrafusal fibres relax simultaneously during the hold period, the firing rate slowed as force decreased. However, as neither force nor fascicle displacement predicted firing rate changes across compliance groups in all stretch types, the complexities of muscle spindle mechanics cannot be perfectly explained by a simple force or length model.

More complex modelling may be necessary to better understand the relationships between multiscale mechanics in the muscle and muscle spindle firing rate. While muscle spindle firing appears more force‐related than fascicle‐displacement‐related during the hold phase, MTU force was only moderately correlated with MTU force here, and the relationship changed between control and SEE trials. Prior models relating MTU force and yank to muscle spindle firing rates in rats and cats (Blum et al., 2020, 2017) are not suited to these data. In rats, the force contribution of extramysial tissue in the muscle has to be estimated from the displacement of the MTU and subtracted from the total MTU force. However, as MTU displacement measurements in these experiments also encompassed the added elastic measurement, it cannot be used as an input to an extramysial tissue model. It is not known if fascicle measurements from sonomicrometry can accurately predict extramysial force, as it may not measure the displacement of the connective tissues carrying the loads (Gillies & Lieber, 2011; Meyer & Lieber, 2011; Meyer & Lieber, 2018). Further, this data‐fitting model is only accurate to the degree that extrafusal and intrafusal forces are approximately proportional. During triangle stretches, the similarity in muscle spindle firing in the second and third stretch is likely due to the intrafusal muscle going slack within the extrafusal muscle (Blum et al., 2017, 2019, 2020; Poppele et al., 1979). Thus, it may be necessary to have a biophysical model to separately predict intrafusal and extrafusal muscle dynamics (Blum et al., 2020) to more accurately predict muscle spindle firing.

Changes in tendon compliance with ageing and disease may have varied and context‐dependent effects on muscle spindle sensory feedback. Increased tendon compliance generally reduced muscle spindle firing, but in complex relationships to muscle mechanics. The changes in muscle spindle firing likely depend on the nature of the movement, in addition to non‐linear properties of the muscle and tendon tissues. Further, the different relationships between muscle fascicle and muscle biomechanics across all stretch types suggest that muscle spindles do not simply encode muscle fascicle length.

## AUTHOR CONTRIBUTIONS

Emily M. Abbott—conception and design, data acquisition and analysis, drafting and revising. Jacob D. Stephens—data analysis and interpretation, drafting and revising. Surabhi N. Simha—data interpretation, drafting and revising. Leo Wood—data analysis, drafting and revising. Paul Nardelli: data acquisition, drafting and revising. Timothy C. Cope—conception and design, drafting and revising. Gregory S. Sawicki—conception and design, data analysis and interpretation, drafting and revising. Lena H. Ting—data analysis and interpretation, drafting and revising. All authors have read and approved the final version of this manuscript and agree to be accountable for all aspects of the work in ensuring that questions related to the accuracy or integrity of any part of the work are appropriately investigated and resolved. All persons designated as authors qualify for authorship, and all those who qualify for authorship are listed.

## CONFLICT OF INTEREST

The authors have declared that no competing interests exist.

## Data Availability

The data that support the findings of this study are openly available in Open Science Framework at osf.io/akqjf. The code used to analyse these data and generate the results is available at: github.com/stephensjake72/SpindleGrant/tree/main/SpindleSpring.
